# Lower Activation in Frontal Cortex and Posterior Cingulate Cortex Observed during Sex Determination Test in Early-Stage Dementia of the Alzheimer Type

**DOI:** 10.3389/fnagi.2017.00156

**Published:** 2017-05-22

**Authors:** Ravi Rajmohan, Ronald C. Anderson, Dan Fang, Austin G. Meyer, Pavis Laengvejkal, Parunyou Julayanont, Greg Hannabas, Kitten Linton, John Culberson, Hafiz Khan, John De Toledo, P. Hemachandra Reddy, Michael W. O’Boyle

**Affiliations:** ^1^Department of Pharmacology and Neuroscience, Texas Tech University Health Sciences CenterLubbock, TX, United States; ^2^Department of Electrical and Computer Engineering, Texas Tech UniversityLubbock, TX, United States; ^3^Department of Human Development and Family Studies, Texas Tech UniversityLubbock, TX, United States; ^4^School of Medicine, Texas Tech University Health Sciences CenterLubbock, TX, United States; ^5^Department of Neurology, Texas Tech University Health Sciences CenterLubbock, TX, United States; ^6^Department of Public Health, Texas Tech University Health Sciences CenterLubbock, TX, United States; ^7^Department of Family Medicine, Texas Tech University Health Sciences CenterLubbock, TX, United States; ^8^Garrison Institute on Aging, Texas Tech University Health Sciences CenterLubbock, TX, United States; ^9^Department of Cell Biology and Biochemistry, Texas Tech University Health Sciences CenterLubbock, TX, United States; ^10^Department of Speech, Language and Hearing Sciences, Texas Tech University Health Sciences CenterLubbock, TX, United States

**Keywords:** Alzheimer, face-processing, neuroimaging, chimeric faces, brain networks, neurodegeneration

## Abstract

Face-labeling refers to the ability to classify faces into social categories. This plays a critical role in human interaction as it serves to define concepts of socially acceptable interpersonal behavior. The purpose of the current study was to characterize, what, if any, impairments in face-labeling are detectable in participants with early-stage clinically diagnosed dementia of the Alzheimer type (CDDAT) through the use of the sex determination test (SDT). In the current study, four (1 female, 3 males) CDDAT and nine (4 females, 5 males) age-matched neurotypicals (NT) completed the SDT using chimeric faces while undergoing BOLD fMRI. It was expected that CDDAT participants would have poor verbal fluency, which would correspond to poor performance on the SDT. This could be explained by decreased activation and connectivity patterns within the fusiform face area (FFA) and anterior cingulate cortex (ACC). DTI was also performed to test the association of pathological deterioration of connectivity in the uncinate fasciculus (UF) and verbally-mediated performance. CDDAT showed lower verbal fluency test (VFT) performance, but VFT was not significantly correlated to SDT and no significant difference was seen between CDDAT and NT for SDT performance as half of the CDDAT performed substantially worse than NT while the other half performed similarly. BOLD fMRI of SDT displayed differences in the left superior frontal gyrus and posterior cingulate cortex (PCC), but not the FFA or ACC. Furthermore, although DTI showed deterioration of the right inferior and superior longitudinal fasciculi, as well as the PCC, it did not demonstrate significant deterioration of UF tracts. Taken together, early-stage CDDAT may represent a common emerging point for the loss of face labeling ability.

## Introduction

Alzheimer disease (AD) is a debilitating disorder marked by a progressive decline in cognitive functions in which the affected individual becomes less capable of understanding the world around them often leading to fear, isolation and depression. Part of this isolation stems from a loss of the ability to classify faces into social categories; a process known as face-labeling. This plays a critical role in human interaction as it serves to define concepts of socially acceptable interpersonal behavior.

In this study, we attempted to determine how verbal impairments may relate to face-labeling. The current study attempted to characterize, what, if any, impairments in face-labeling are detectable in participants with early-stage clinically diagnosed dementia of the Alzheimer type (CDDAT) through the use of the sex determination test (SDT). Psychological research on categorical grouping of faces previously hypothesized that this was likely a verbally-mediated process (Chance and Goldstein, 1976; Perdue et al., 1990; Otten and Wentura, 1999). More recent work, however, has shifted emphasis away from verbal-labels in favor of visuospatial constructs such as “face space” upon which facial characteristics are imposed and the composite of which are then assigned to a category (Valentine, 1991). This view has since gained ground through findings from computer-based geometric modeling (Valentine, 2001) and neuroimaging (Oruç et al., 2011; Contreras et al., 2013). These observations suggest that social categorization is likely a two-step process: one in which the visuospatial components of a face must be decoded and grouped with similar faces, and then a verbal-label is generated to describe to which group the face belongs, thereby making the verbal component significantly more removed than previously thought.

Existing clinical work, however, has established that verbal impairments occur early within the course of AD (Chobor and Brown, 1990; Locascio et al., 1995; Henry et al., 2004) as do impairments in gender discrimination (Rombouts et al., 2005). These impairments are congruent with the pathological progression of the disease (Braak et al., 2006; Jack et al., 2008) and we therefore sought to identify what functional and connectivity-based alterations may underlie this pathology.

To this end, the ability to identify the sex and race of faces was specifically localized to activity within the fusiform face area (FFA) in neurotypicals (NT) by Contreras et al. (2013). Potential damage to this region is of particular interest in the context of AD, given the aforementioned pathology within the temporal lobes (Braak et al., 2006) and consistently observed verbal impairments (Henry et al., 2004).

We interpret these findings to suggest that during early-stage AD, FFA activity is still intact, but the decreased temporal derivative in the anterior cingulate cortex (ACC) observed by Rombouts et al. (2005) hints at a developing inability to successfully discriminate between genders. It is important to note, however, that neither of these studies used chimeric face tests (CFTs) and the gender discrimination task (GDT) is slightly, but fundamentally, different from our SDT.

Therefore, we hypothesized that the increased demand on the FFA caused by presenting CFTs, which are substantially harder to process than natural faces, would lead to an observable difference in FFA activity and task performance between our CDDATs and NTs if one were readily existent. In order to further stress this difference, the SDT asked participants to determine if the two faces were of the same sex, and not which sex they were (as the GDT did). By doing so, we placed greater emphasis on making an executive decision related to a specific verbal-label (i.e., greater emphasis is placed on a participant’s ability to recognize if two faces are either “men, women, or one of each”) with less emphasis on their ability to generate verbally-mediated labels (e.g., “men, women, or both”). This, in turn, would likely flush out differences in ACC activity (Bush et al., 2000), similar to Rombouts et al. (2005), thereby giving us the opportunity to assess the relative importance of FFA and ACC activity within a single task.

Lastly, verbal skill tests have been used to define the progression of cognitive decline in AD (Verma and Howard, 2012). Pineda-Pardo et al. (2014) correlated fractional anisotropy (FA) values of several known speech areas to performance on the verbal fluency test (VFT) in patients with mild cognitive impairment of the amnestic type (aMCI), a common precursor to AD, showing that there may be structural as well as functional alterations underlying this deterioration. Therefore, establishing what association may exist between face-processing and verbal skills, as well as their underlying circuits of activity and connectivity, is of great importance in order to assess their potential for assisting in the early detection of this disease.

fMRI studies on aMCI patients routinely focused on memory and showed increased activation in individuals with the mildest forms of impairment while those who showed decreased activity were more likely to be in the most severe end of the spectrum (Dickerson et al., 2005; Celone et al., 2006; Johnson et al., 2006; Trivedi et al., 2008). This led Risacher and Saykin (2013) to conclude that the initial increases observed in the early-stages of impairment represent attempted compensation while the decreased activity seen upon greater impairment is due to deterioration of the system after it is no longer capable of compensating.

This pattern of early compensation followed by decreased activity is repeated in AD patients. Pariente et al. (2005) noted increased activation during episodic memory encoding and recall in the posterior cingulate cortex (PCC), precuneus, parietal lobe and frontal lobe in early-stage AD patients with a mean Mini Mental State Exam (MMSE) score of 25 ± 1.8. Grady et al. (2003), Dickerson et al. (2005), Celone et al. (2006) and Zhou et al. (2008) on the other hand, observed decreased or even absent activation relative to NTs in the same regions in early-stage AD patients with mean MMSE scores of 21.1 ± 3.1, 22 ± 5, 21.3 ± 2.7, respectively, suggesting that the differences in observations are congruent with previous findings in aMCI patients with regard to progression of symptoms. In particular, the default mode network (DMN; which includes the PCC, medial parietal lobe and medial frontal lobe) shows decreased activity at rest, decreased connectivity and reduced deactivation upon task initiation in AD patients (Grady et al., 2003; Greicius et al., 2004; Buckner et al., 2005; Celone et al., 2006).

It is important to note that this significant difference in neuroimaging findings occurred in the same clinically specified stage (i.e., early-stage AD) as defined by MMSE scores (between 26–21, National Collaborating Centre for Mental Health (UK), 2007) demonstrating the importance of integrating neuroimaging results with clinical observations. For a comprehensive review of AD discoveries through neuroimaging, see Risacher and Saykin (2013).

Of equal importance is the understanding of how white matter (WM) tracts are affected by the disease. Previous DTI studies have shown reduced FA in the parietal and temporal WM, the corpus callosum and the posterior cingulum fibers (Medina and Gaviria, 2008). WM alterations appear to parallel the previously discussed gray matter (GM) changes in that cortical abnormalities are greater in posterior brain regions relative to anterior regions at the early-stages of AD (Arnold et al., 1991; Braak and Braak, 1995). When the disease progresses, the neurofibrillary pathology advances from limbic to frontal structures, into higher-order association cortices, and finally affects primary sensorimotor areas, which correlates with the clinical manifestations of AD. DTI-based tractography studies (Fellgiebel et al., 2005) and whole-brain DTI studies (Medina et al., 2006; Rose et al., 2006; Zhang et al., 2007) have consistently shown that fibers located deep in the posterior WM (e.g., the superior longitudinal fasciculus and the posterior cingulum bundle) are affected in patients with AD and aMCI. Bartzokis et al. (2003, 2004) and Bartzokis (2004) have proposed that this may occur because as brain development takes place, later myelinated regions (cortical association areas) have fewer oligodendrocytes supporting greater number of axons compared with earlier myelinated regions. The oligodendrocytes in the cortical association areas therefore have higher metabolic demands in order to maintain their widely distributed axons, making them more susceptible to pathological processes. The DTI findings of decreased WM integrity in later myelinated regions at the onset of AD support this “reversed demyelination” construct (Medina and Gaviria, 2008).

Following the abundance of evidence of WM compromise in AD, a pilot study using this approach found that both aMCI and AD groups showed significant reductions in *FA* within the temporal lobes (Huang et al., 2007) and regional estimations of WM integrity been linked to MMSE scores (Folstein et al., 1975), with lower scores being associated with decreased WM integrity in cerebral posterior regions of AD subjects (Rose et al., 2000; Bozzali et al., 2002; Yoshiura et al., 2002; Duan et al., 2006).

Research on aMCI populations using neuropsychological tests of declarative memory extend this trend as they have demonstrated significant correlations between declining performance and decreases in posterior WM *FA*, particularly in the posterior cingulum bundles (Fellgiebel et al., 2005, 2008; Rose et al., 2006). Harking back to the proposal of Bartzokis et al. (2004), disruptions in transcortical connectivity may serve as early contributors to the pathophysiology of dementia as the observed WM deteriorations were embedded beneath cortical GM that is often affected early within the disease course. For a more in-depth review of DTI findings in AD, see Medina and Gaviria (2008).

Although memory deficits are a hallmark characteristic of AD, there may be little to gain from testing face-processing related to familiarity that isn’t already known (Sperling et al., 2003; Golby et al., 2005; Winchester, 2009; Donix et al., 2013). On the other hand, fundamentals of face-processing (e.g., categorical labeling), as well as its relation to verbal skill sets, are areas that have received little attention (Job, 2012) despite their crucial role in daily life and therefore warrant further investigation.

The use of neuropsychological tests in combination with neuroimaging techniques stresses the importance of attempting to integrate pathological observations with clinical symptoms. In doing so, we are able to reinforce findings from either end of the spectrum as well as more efficiently develop our understanding of the disease and what measures may be taken to help those afflicted. In order to investigate a cognitive operation as complex as face labeling, it will be necessary to use appropriate tests that can isolate it as a specific subdivision of face-processing. This is particularly important when performing an fMRI investigation, as some studies suggest that processing and recognition occur automatically (Vuilleumier and Schwartz, 2001) while others say this can occur within 100 ms of being presented with a face (Batty and Taylor, 2003). If such factors are not properly accounted for, it would be very difficult to remove these confounds from the data as the canonical hemodynamic response curve suggests that blood flow in response to neuronal activity peaks between 4 s–6 s after the presented stimuli, making it far too slow to tease out processes that are nearly instantaneous by comparison (Poldrack et al., 2011).

To this end, a modified version of a specific neuropsychological test will be of particular value when investigating face labeling in AD. The CFT was originally developed by Levy et al. (1983) “to index functional cerebral asymmetry for processing facial characteristics”. Through neuroimaging and lesion studies the FFA has been identified as being highly involved in many face-processing tasks (Kanwisher et al., 1997). In 2008 an fMRI study by Yovel et al. (2008) on face-processing concluded that FFA asymmetry is a highly stable individual characteristic that underlies the well-established left-visual-field superiority for face recognition (Yovel et al., 2008), making it an ideal region of interest when investigating the effects of AD on face-processing.

CDDAT participants were expected to show poor performance on the SDT, which could be explained by decreased activation and connectivity patterns within the FFA (Contreras et al., 2013) and ACC (Rombouts et al., 2005). This was expected to correlate to poor performance on the VFT. This would have established that sex determination is affected in early-stage AD (as was previously demonstrated by Rombouts et al., 2005, but not using Chimeric Faces). Said decrease in performance would likely be due to altered brain activity/connectivity patterns within the FFA, which may correlate sex determination and verbal fluency performance, thereby strengthening the notion that sex determination is a verbally-mediated face labeling process.

## Materials and Methods

### Participant Identification and Selection

The University Medical Center Departments of Neurology, Family medicine, and Geriatrics saw 171 patients for complaints of “memory problems” or “dementia” by during a 6-month period (November 2015–April 2016). Hundred of these patients were determined to have AD or its precursor, aMCI, in accordance with the guidelines outlined by McKhann et al. (2011) for “dementia due to AD” or “mild cognitive impairment due to AD” (Albert et al., 2011) by physician assessment. In accordance with National Institute for health Care and Excellence guidelines (National Collaborating Centre for Mental Health (UK), 2007), those in the possible early-stage AD category were selected based on an MMSE score of 26–21. Twenty of these patients were determined to fit our inclusion/exclusion criteria; four of whom agreed to participate. All CDDAT participants had an existing medical MRI scan interpreted by a radiologist within the last 5 years prior to the study. These individuals were grouped into the CDDAT category. Detailed inclusion/exclusion criteria for the CDDAT group are listed below:

Inclusion Criteria
• The diagnosis of AD was made in line with international diagnostic criteria (Albert et al., 2011; McKhann et al., 2011) on the basis of clinical history, neurologic, psychiatric, neuropsychological evaluation and hematologic screening for dementia (including liver and kidney function, thyroid function, folic acid, and B12 vitamin levels, neoplastic markers), electroencephalogram, carotid ultrasonography and neuroimaging studies (computed tomography and/or magnetic resonance) as determined by the attending physician.
• Mean general cognitive decline was evaluated with the MMSE (Folstein et al., 1975).
• All participants gave their informed consent to participate in the study, which received approval from Texas Tech University Human Protections Internal Review Board.
Exclusion Criteria
• Inability to understand the instructions from the principal investigator (PI) or research conductors.
• History of substance abuse.
• History of neurological deficits not related to Alzheimer disease.
• Inability or refusal to give informed consent.
• Motor, visual, or hearing impairment that precluded ability to perform cognitive testing assessments.
• Failure to pass the MRI safety checklist guidelines.

Ten age-matched cognitively normal participants (5 males, 5 females), determined by physician assessment, were recruited from a nearby senior living center and were grouped into the NT category. NTs were required to have an MMSE score >27 and meet all inclusion and exclusion criteria stated above, except for that concerning the existence of Alzheimer-related pathology. One male participant was retroactively removed after receiving a diagnosis of normal pressure hydrocephalus.

All control participants are also patients followed by our physicians, not merely elderly individuals from the community who have been screened using an MMSE. Although our Human Protections protocol prevents us from accessing or further commenting on medical information on these patients other than their MMSE scores, our physicians selected patients for both the control and experimental group in accordance with the accepted international guidelines outlined by Albert et al. (2011) for the diagnosis of mild cognitive impairment. An example of the fact that the control individuals were appropriately screened for possible neurological deficits is evidenced by the retroactive removal of one of the NTs after a suspicion by clinical evaluation led to a clinical MRI scan which demonstrated normal pressure hydrocephalus. The physicians promptly informed us of the finding and the participant was removed from the study.

Participants were informed that their participation was voluntary and they may withdraw from the study at any time and that their refusal to participate would have no impact on their level of care. This study was approved by the Texas Tech University Human Protections Internal Review Board. The subject was given a copy of the informed consent form and each section of the informed consent form was verbally reviewed. Subjects were given time to ask any questions they may have. If the subject verbally agreed to participation, the subject was asked to briefly summarize their understanding of the research project and what they were agreeing to do. Any minor misconceptions were corrected and the subject was again allowed to verbally decide. After this process, the subject was allowed to sign the consent form and received a copy of the signed consent form to keep. Written informed Consent was obtained in the presence of the primary care giver.

#### Sample Size

Henry et al. (2004) calculated an effect size of *r* = 0.73 for a test of semantic fluency in patients with Dementia of the Alzheimer Type (DAT). That effect size r is equivalent to Cohen’s *d* = 2.14. To achieve 80% power, we needed a minimum sample size of *n* = 5. Given our heterogeneous group sizes of four disease and nine control, we were able to achieve 90% power.

#### Participant Demographics

We recruited four right-handed Caucasian patients (3 males, 1 female) with a diagnosis of aMCI or early-stage AD between the ages of 73–93 (*median age* 83.5 ± 8.4) with MMSE scores between 26–23 (*median of* 24.5 ± 1.3). Two of the males (MMSE scores of 26 and 25) had an existing diagnosis of “mild cognitive impairment due to AD” (Albert et al., 2011). The remaining male and female had an existing diagnosis of “dementia due to AD” (MMSE scores of 24 and 23, respectively; McKhann et al., 2011). Nine (8 right-handed and 1 non-right-hand-dominant) cognitively normal Caucasian participants (4 males, 5 females; all with MMSE scores of 30) between the ages of 79–91 (*median age* 80 ± 3.8) also participated. Handedness was determined using the Edinburgh Handedness Inventory-revised (EHI-r; Williams, 1986). There was no significant difference in median age (*p* = 0.582), but there was an observable difference in median MMSE score for the two groups (*p* = 0.011).

While there may be some concern given our lack of equal sex-distribution (1 female and 3 males) within the CDDAT group, no differences in performance were discernable based on sex within either our NT or CDDAT groups across any cognitive test (VFT or SDT) for either accuracy or reaction time (RT; data not shown) nor for within-group sex-based contrast mapping for fMRI or DTI (data not shown). A summary of participant demographics is given in Table 1.

**Table 1:** **Participant demographics**.

Group	Age (median ± SD)	Sex	Handedness	Race	MMSE (median ± SD)
CDDAT (***n*** = 4)	83 ± 8	1 F, 3 M	4 R	4 CA	24.5 ± 1.3
NT (***n*** = 9)	80 ± 4	5 F, 4 M	8 R, 1 nR	9 CA	30 ± 0

*CDDAT, clinically diagnosed dementia of the Alzheimer type; NT, neurotypical; F, female; M, male; R, right-handed; nR, non-right-handed; CA, Caucasian*.

### Administration of Verbal Fluency Test

Participants were asked to list all the names of animals they could recite in 1 min. The same procedure was then repeated for names of fruit and finally for colors. Audio recordings were taken of the verbal tasks. Scores were expressed as the sum of the three tasks.

### Imaging Methodology and Analyses

#### Stimulus Presentation and Participant Response

**Figure 1:**
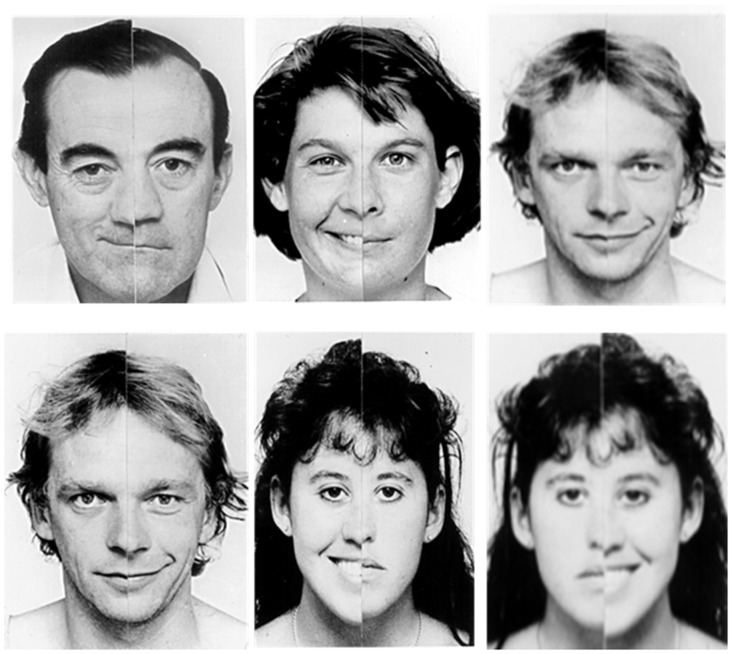
**Examples of the sex determination test (SDT)**. SDT examples in response to the question: “ARE THE TWO PEOPLE OF THE SAME SEX?” (left) “yes”; two males (center) “yes”; two females (right) “no”; male (top) and female (bottom).

After completing the VFT and Rey Osterrieth Complex Figure B (ROCFB), all participants were briefly trained on the SDT before entering the scanner to ensure task comprehension by allowing them to practice on a single example trial of each condition type (Rajmohan, 2016). Chimeric faces (Mattingley et al., 1993) were presented in an event-related design using Eprime 2.0. Presentation order was counterbalanced across participants using a Latin square design. The question “Are the two people of the same sex?” was displayed on the screen inside the scanner for 4 s. A set of 40 trials were randomized and displayed for 4 s each with a jittered inter-stimulus interval (ISI) randomized for five time points between 800 ms and 1200 ms, by 100 ms apiece. Participants responded using a fiber optic controller held in the right-hand where button 1 was pressed by the index finger and button 2 was pressed by the middle finger. Participants pressed one of two buttons to indicate Yes (button 1) or No (button 2). An example of the possible stimulus presentations is given in Figure 1.

#### Scanning Parameters

All images were acquired with a 3T Siemens MR system (Skyra, Germany) at the Texas Tech Neuroimaging Institute. The T1 anatomic scan parameters were: TR: 1900 ms; TE: 2.49 ms; FOV: 240; Flip angle: 9, Voxel size = 0.9 × 0.9 × 0.9 mm; slice number: 192. The fMRI parameters were: TR: 2500 ms; TE: 20.0 ms; FOV: 231; Flip angle: 75, Voxel size = 2.5 × 2.5 × 3.0 mm; slice number: 41. The DTI parameters were: TR: 5000 ms; TE: 95 ms; FOV: 220; B/W: 1562, Voxel size = 1.7 × 1.7 × 4.0 mm; slice number: 32.

#### Image Preprocessing

##### fMRI

Image preprocessing steps included removing non-brain structures by Brain Extraction Tool (BET), motion correction by using Motion Correction for FSL Linear Registration Tool (MCFLIRT), temporal high-pass filtering with a cutoff period of 24 s, spatial smoothing with a 5 mm Gaussian full width, half maximum (FWHM) algorithm, and co-registering of the functional images to the high resolution T1 structure images in their native space using boundary border registration(BBR) and FSL Linear Registration Tool (FLIRT; Jenkinson and Smith, 2001; Jenkinson et al., 2002) at 12 degrees of freedom to the Korean Normal Elderly (KNE96; Lee et al., 2016) standard brain space.

##### DTI

FSL Diffusion Toolbox 3.0 (FDT) from FMRIB Software Library (FSL 5.0.5) was used to complete the construction of and preprocessing for all anatomical brain networks for all subjects. It processed DICOM/NIfTI files into diffusion metrics (e.g., *FA*) that were ready for statistical analysis at the voxel-level after performing corrections for image alignment and artifact clean-up (Top-Up) and local field distortions (eddy current correction; Smith et al., 2004).

#### Image Processing

##### fMRI

fMRI data processing was carried out using FMRI Expert Analysis Tool (FEAT) Version 6.00, part of FMRIB’s Software Library^1^ (FSL). The time series for the behavioral events were analyzed for the following conditions.

For “presentation of stimuli scenarios” (i.e., when the participant was shown two male faces, two female faces, or one of each)—Brain activity from the first 2 s following the presentation of a stimulus was recorded for each trial. In the event a participant responded within <2 s, the interval between the presentation of the stimulus and 250 ms before the response was used for the recording interval. This was done to avoid brain activity artifacts related to the button press. Instances where a participant made no response before the presentation of the next stimulus were discarded.

For “participant response” scenarios (when the participant chose “yes” or “no” in response to the question: “Are the two people of the same sex?”)—Brain activity from the first 2 s following the press of a button was recorded for each trial. In the event a participant responded within <2 s before the presentation of the next stimulus, that interval was used for the recording time. Instances where a participant made no response before the presentation of the next stimulus were discarded.

A summary of fMRI contrasts is given in Table 2. A total of five subject-level contrast maps were created for all subjects using threshold free cluster enhancement (TFCE) of *z* > 2.3 and a cluster corrected significance threshold of *p* < 0.05. These modeled time series were convolved with the double gamma hemodynamic response function (dg-HRF), which was modeled from a combination of Gaussian functions. Group-level contrast maps were created using FMRIB’s Local Analysis of Mixed Effects (FLAME1). The thresholds for group level activation maps were created using TFCE of *z* > 1.5 and a cluster-corrected significance threshold of *p* < 0.05. The exact regions of brain activity were determined using the KNE96 coordinate space and Harvard-Oxford cortical and subcortical structural atlases.

**Table 2:** **Summary of fMRI contrasts**.

Task type	Option 1	Option 2	Option 3
SDT-presentation of stimuli:	Two male faces	Two female faces	One of each
SDT-choice of the participant:	Yes	No	–

*A total of five subject-level contrast maps were created for all subjects. SDT, sex determination test*.

##### DTI

Voxel-based group differences were calculated for the *FA* images using Tract-Based Spatial Statistics (TBSS; Smith et al., 2006; Smith and Nichols, 2009; Cheon et al., 2011). TBSS linearly registered individual *FA* images in native space and then to the *FA* template via the *flirt* command of FSL. The resultant warping transformations were then used to convert images of diffusion (i.e., *FA*) to MNI152 (Montreal Neuroimaging Institute) space with a spatial resolution of 1 × 1 × 1 mm. For statistical inference, including correction for multiple comparisons, permutation testing was used (Nichols and Holmes, 2002; Cheon et al., 2011) as implemented by RANDOMISE of the FSL software package. Five-hundred permutations were performed for significant group differences at a threshold of 0.2; corresponding to *p* < 0.05, corrected for multiple comparisons using TFCE (Smith and Nichols, 2009; Cheon et al., 2011). WM tracts were identified using the Johns-Hopkins University ICBM-DTI-81 WM labels and probabilities of tract accuracy were assessed using the Johns-Hopkins University WM Tractography atlas.

### Behavioral Methodology and Analyses

All statistical calculations for behavioral analyses were performed using Rstudio Desktop (version 0.99.896, Rstudio, Inc., Boston, MA, USA).

#### Comparison of Test Performance Amongst Study Groups

Mann-Whitney *U*-tests with false discovery rate (FDR) corrections of α = 0.05 for multiple comparisons to reach significance at *p* < 0.05 were used to evaluate differences in test performance between the study groups. A one-sample *t*-test with mu = 30 and two-sample *U*-test were used to calculate the group difference in MMSE score given that the variance for the NT group was 0. Both tests revealed a significant result. Additionally, the zero variance for the NT group on the MMSE is within reason for cognitively normal elderly individuals, as persons without cognitive deficits may be expected to receive a perfect score (30/30) as was seen here (Folstein et al., 1975).

#### Correlation of CFTs to Cognitive Test Performance

Pearson correlation coefficients, with FDR corrections of α = 0.05 for multiple comparisons to reach significance at *p* < 0.05, were used to correlate SDT performance to VFT performance.

#### Assessing Tests as Classifiers

Fischer Exact test, with FDR corrections of α = 0.05 for multiple comparisons to reach significance at *p* < 0.05, were used to determine strength of each test as a classifier between CDDAT and NT.

## Results

### Behavioral Data

**Figure 2:**
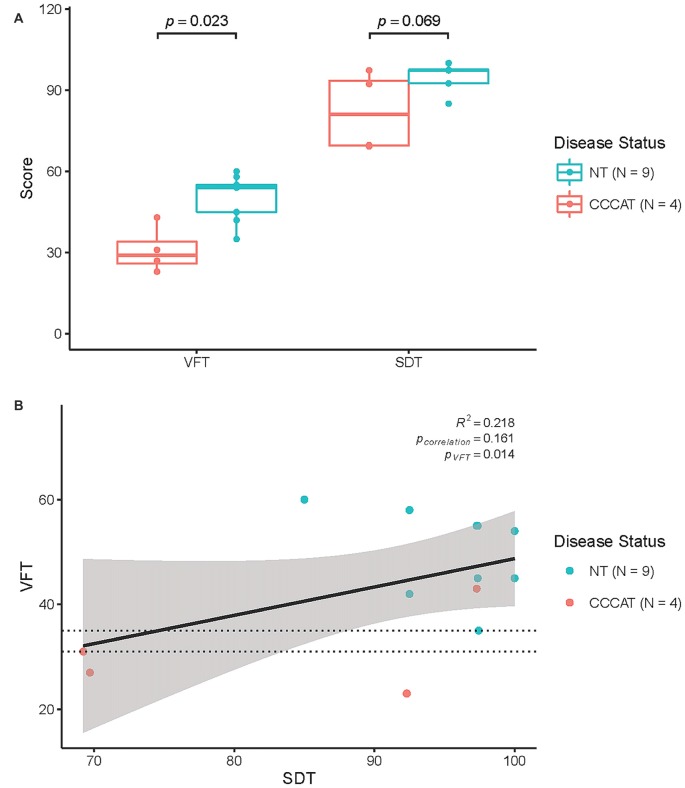
**Behavioral results. (A)** Results of Verbal Fluency Test (VFT) and SDT performance. **(B)** Correlation of VFT and SDT performance. NT, Neurotypicals; CDDAT, Clinically diagnosed dementia of the Alzheimer type. Thickened line is VFT classifier score at 31–35; *p* = 0.014.

Consistent with previous studies, the median VFT score was significantly different between NTs and CDDATs (54 ± 8 vs. 29 ± 9, *respectively; p* = 0.023, *Cohen’s d* = 3.01, *r-equivalent effect size* = 0.833). VFT was a successful classifier at an optimal score range of 31–35 (*p* = 0.014). Additionally, while SDT performance was not significantly different between median NT and CDDAT scores (97.4 ± 5.0 vs. 81.0 ± 14.8%; *p* = 0.069, *Cohen’s d* = 2.05, *r-equivalent effect size* = 0.715), this was due to half of the CDDAT participants performing substantially worse than NT (*70.0 ± 0.3%*), while the other half performed very similar (*94.8 ± 3.5%)*. As such, SDT was not a successful classifier at any score (optimal score range 69–85). CDDAT RT, however, was not significantly different from NTs for SDT (1757 ms vs. 2007 ± 457 ms, *not shown*), suggesting intact comprehension of task instructions despite their varied performance. We found no connection between verbal ability and face-labeling (*VFT ~ SDT; R*^2^ = 0.218, *p* = 0.161). Behavioral results are summarized in Figure 2 and Table 3.

**Table 3:** **Summary of Verbal Fluency Test (VFT) and Sex Determination Test (SDT) results**.

Participant ID	VFT	SDT (%)
NT 01	54	100
NT 02	55	97.2973
NT 04	45	100
NT 05	42	92.5
NT 06	58	92.5
NT 07	35	97.4359
NT 08	55	97.36842
NT 09	60	85
NT 10	45	97.36842
CDDAT 01	23	92.30769
CDDAT 02	31	69.23077
CDDAT 03	27	69.69697
CDDAT 04	43	97.2973

*NT, Neurotypical; CDDAT, Clinically Diagnosed Dementia of the Alzheimer Type; VFT scores are reported as total number of words*.

### Imaging Data

#### fMRI Results

**Figure 3:**
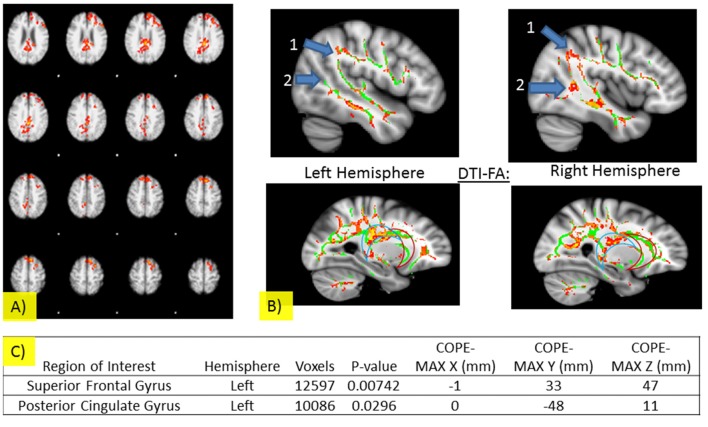
**(A)** Significant fMRI activations for SDT when participants responded “yes” in left-Superior Frontal Gyrus and left-Posterior cingulate cortex (PCC; threshold free cluster enhancement, threshold free cluster enhancement, TFCE of *z* > 1.5 and a cluster-corrected significance threshold of *p* < 0.05). **(B)** Pertinent DTI results for the SDT. Significant differences were observed for the superior longitudinal fasciculi (number 1) and inferior longitudinal fasciculi (number 2) for the left (top left) and right (top right) hemispheres and for the PCC (blue crescent outline), but not the anterior cingulate cortex (ACC; red crescent outline) for the left (bottom left) and right (bottom right) hemispheres. For Figures: areas where NT brain activity > CDDAT brain activity are shown in red and CDDAT brain activity > NT brain activity are shown in blue. For fMRI figures: areas where NT brain activity > CDDAT brain activity are shown in red. For DTI figures: areas where NT > CDDAT for fractional anisotropy (FA) measures are shown in red. Green lines represent the FA skeleton. NT, Neurotypicals; CDDAT, Clinically Diagnosed Dementia of the Alzheimer Type. **(C)** Significant fMRI activations for SDT when participants responded “yes” are listed for the NT > CDDAT contrast (TFCE of *z* > 1.5 and a cluster-corrected significance threshold of *p* < 0.05). No significant fMRI activations for SDT were seen for the CDDAT > NT contrast.

Imaging results are summarized in Figure 3 and Table 3. There were no significant differences in areas of activation between the NT and CDDAT groups upon stimulus presentation regardless of the sex of the faces, indicating no fundamental difference in perception of stimuli. NTs, however, were shown to have greater activity in the left superior frontal gyrus (l-SFG) and left posterior cingulate cortex (l-PCC) when responding “yes” to the question “Are the two people of the same sex?” CDDAT did not show higher values in any areas for any contrasts.

#### DTI Results

NTs showed higher *FA* values than CDDATS in the right inferior longitudinal fasciculus (r-ILF), right posterior thalamic radiations (r-PTR) and the bilateral PCC (b-PCC) and superior longitudinal fasciculi (b-SLF). For the SLF, differences between NTs and CDDATs were greater in the left than the right hemisphere. No tracts showed higher *FA* values for the CDDAT group than NT group. *FA* measures did not demonstrate significant differences throughout the uncinate fasciculi (UF). However, CDDATs were noted to have less frontotemporal connectivity than NTs via lower *FA* values throughout the r-ILF and r-SLF as well as the b-PCC, but not ACC. Imaging results are summarized in Figure 3.

## Discussion

### Significance of Behavioral Findings

In this study, we chose to use the VFT given its previous use by Luzzi et al. (2007) and Pineda-Pardo et al. (2014) and its validation by Robert et al. (2003), which gave us confidence in its suitability as a measure of verbal ability. Although the VFT was a successful classifier between CDDAT and NTs, we were unable to establish a direct correlation between VFT and SDT performance. Furthermore, SDT performance was not significantly lower in CDDATs compared to NTs. Given the dichotomous performance of our CDDAT group on the SDT, however, along with their significantly lower performance on the VFT, we reason that, while the SDT may not directly correlate with verbal proficiency, it may still represent an accurate assessment of face-labeling ability. The discrepancy between VFT and SDT performance can therefore be explained as the verbally-mediated component of the SDT (i.e., observing two faces and determining whether they are a “man” or “woman” and then determining if they are “same” or “different”) being less demanding of verbal ability than the VFT, which requires spontaneous word generation within the confines of an abstract category. Therefore, it is reasonable to expect impairments in verbally-mediated processes to manifest sooner and more drastically through the VFT than the SDT. Support for this argument may be inferred from our behavioral results, which show that, while SDT score varies greatly between the highest and lowest (standard deviation of 14.8%) performance by CDDAT, there is less change in VFT (standard deviation of 9%).

In spite of this, the SDT still has clinical merit for individuals with suspected dementia as it mirrors a more realistic point of assessment (i.e., can patients determine the sex of a person they are talking to?) in relation to social interaction and activities of daily living than the word-finding nature of the VFT. Lastly, the split in CDDAT performance gives us confidence that this stage of the disease does, in fact, represent the initial stages of face-labeling deterioration given that Luzzi et al. (2007) observed significant impairment by the moderate-stage in AD patients.

### Significance of fMRI Findings

Our fMRI findings further support this notion, in that, they did not show an observable difference in FFA or temporal lobe activity between CDDAT and NTs, even when specifically contrasting the subgroup of low-performing CDDAT individuals (data not shown). While it is possible that the lack of difference in findings within the FFA and temporal lobes for the subgroup contrast map may be attributable to a lack of power due to small sample size (*n* = 2; *design efficiency of* 0.447), their absence from the total group contrast map, in the presence of lower activity within the l-PCC and l-SFG, demonstrates that impairments in verbally-mediated label generation or fundamental aspects of face-processing were at the very least less pronounced than differences in attention and self-referential decision making processes. This is consistent with common associations of both the l-PCC and l-SFG that has been established by previous work (Maddock et al., 2003; Goldberg et al., 2006; Leech and Sharp, 2014).

Additionally, while decreases in PCC activity are commonly associated with AD (Leech and Sharp, 2014), the lack of this observation within the CFT and EVDT for our CDDAT participants (Rajmohan, 2016) highlights its exclusiveness to the SDT, and is likely a reflection of the intended demanding nature of the task as previously discussed. In spite of this, no difference was observed in either the ACC or the FFA. This leads us to conclude that although their verbal skills are declining, early-stage CDDATs are still capable of a similar level of verbal-labeling required for this task and have fundamentally intact face-processing abilities. Instead, impairments in executive decision-making likely explain the discrepancies in scores seen here, which is consistent with the findings of Rombouts et al. (2005).

### Significance of DTI Findings

The lack of difference in UF tract integrity, as assessed by *FA*, contradicts our initial hypothesis, but strengthens the notion that the connections most crucial to face labeling are largely intact. However, the lower *FA* values in the r-ILF, r-SLF and PCC observed in the CDDAT group suggest deterioration of right-sided frontotemporal connectivity and posterior cingulate fiber bundles, respectively. This deterioration in connectivity would likely explain the simultaneously observed decrease in activity in the l-PCC and l-SFG during CDDAT response as a loss in connectivity reduces system efficiency, which in turn manifests as decreased task-related signal activity. Support for this hypothesis also comes from our simultaneous investigations of CFT and EVDT performance in these participants, which showed that behavioral impairments correlated to deterioration of WM tracts, but no observable differences in brain activity (Rajmohan, 2016). These findings give insight into how the deterioration of WM tracts may predate decreases in brain activity in AD. It is therefore of great interest to see how decline in verbal fluency and face-labeling may occur in tandem with the deterioration of WM tracts and decreasing brain activity as the disease progresses.

### Limitations

Since the etiology of AD is unknown, it is possible that it is the culmination of many factors that may vary in both course and presentation. Therefore, the findings noted in this study population may not extend to all affected individuals due to issues of hereditary or environment. We also acknowledge that aMCI has an insidious course and often goes undetected for years in the absence of rigorous neuropsychiatric testing. It has been noted that in 6 years’ time about 70% of aMCI patients develop AD (Mauri et al., 2012). Therefore, while we cannot rule out the possibility that participants in our control group may inevitably progress to aMCI, they did not meet the accepted international diagnostic criteria for this diagnosis at the time of the study.

Other potential cofounders could be differences in the demographics and comorbidities in the two groups. Due to restrictions related to the approved Human Protections Board protocol, we do not have any additional medical or demographic information (e.g., marital status, list of medications or medical comorbidities that are not of a neurologic or psychiatric nature) on our participants than what is currently described. Although we found statistically significant differences in both connectivity and activation patterns between our CDDAT and NT participants, it is important to acknowledge that this is a pilot study that is limited by its small sample size. As such, it is imperative to demonstrate its reproducibility on a larger scale.

While it may seem odd that the areas of WM deterioration do not directly overlap with those of task-related decrease in activity for all noted instances, this may be because the observable areas of decreased activity are those most crucial for performing the task at hand (in this case, left-hemispheric areas, given the indirect verbal component of this task) and are therefore the first to be detectable in a compromised system. We cannot rule out the possibility that differences in performance are based on dysfunctions in processing facial characteristics that predate verbal labeling based solely on the lack of differences in activity observed through the “stimulus-presentation” contrast map. Therefore, early-stage CDDATs may perceive the sex of faces in a different manner than NTs. A task that focused more specifically on the overlapping regions of interest may more directly demonstrate a correlation between tract integrity and decreased activity, but such a task would likely be of less clinical significance than the SDT.

Additionally, while performance on the VFT was consistent with previous reports (Henry et al., 2004), our SDT findings differed from those of Rombouts et al. (2005). This is attributable to several factors. First, our CDDAT population consisted of a milder stage of impairment (*median MMSE score of* 24.5 ± 1.3 vs. 22.5 ± 2.2). Second, the split in CDDAT performance previously mentioned makes our subgroups (*n* = 2) too small to achieve significant power in spite of the >20% difference in performance, even though this was over twice the effect size (9%) observed by Rombouts et al. (2005) given that their study had six times as many participants. Finally, the SDT is a similar, yet fundamentally different, task from the GDT used by Rombouts et al. (2005) in that the SDT places greater emphasis on decision-making related to the grouping of chimeric faces based on sex, whereas the GDT more directly asks a participant to assign a face to its appropriate sex. All of these factors combine to allow us to suggest that these findings are, in fact, in congruence with those of Rombouts et al. (2005), but merely represent a different circumstance.

Finally, the inability to find a significant difference between our NT and CDDAT groups for the SDT does not necessarily mean that impairments are not present at this time. Instead, it indicates that such a deficit, if it exists, is less severe than that of verbal fluency, which currently represents the most consistent and pronounced clinical manifestation of this disease (Henry et al., 2004) and was observed within this study.

## Conclusion

Given that declines in verbal fluency are already present by this stage, one way to reduce confusion is to use simple, concrete words. We therefore suggest the use of binary categorization (e.g., “yes” or “no”) strategies in place of word labels when dealing with early-stage AD patients. Performance by our CDDAT participants on the SDT suggests that they are able to perform “same/different” classifications more readily than verbally-mediated tasks that emphasize word generation. Additionally, given that our fMRI data suggest greater strain upon verbally-mediated decision making, avoid excessive open-ended questioning in favor of directed questions with discrete responses. By following these simple strategies, we may ease the frustration experienced by both patients and their care-givers.

Although we highlighted several concerns related to how the findings of this investigation may fit into the larger literature, these seemingly limiting differences in many ways provide us with a great deal of new information. By noting the split in CDDAT performance in this study within the context of the performance for MCI (which were shown to perform as well as NTs) and AD patients (who were impaired on the task) from Rombouts et al. (2005), we can be quite confident that we have identified the initial stages of face-labeling impairment. From this, we can infer that: (1) verbal fluency decline predates loss of face-labeling ability; (2) differences in brain activity patterns, along with a lack of difference in UF tracts within the early-stages of face-labeling impairment suggest a more crucial role for executive function than verbal generation when performing the SDT; and (3) destruction of WM tracts may predate GM changes, as observed by the deterioration of frontotemporal WM connections; as such, greater emphasis should be placed on the anatomical and cellular pathology of WM in the study of AD pathogenesis as was suggested by Brun and Englund (1986) nearly 30 years ago.

## Author Contributions

RR created the experimental design, ran the study, analyzed the data, interpreted the results and wrote the manuscript. RCA and DF consulted on neuroimaging design, analysis and interpretation. AGM and HK consulted on statistical analysis and interpretation. PL, PJ, GH, KL, JC and JT consulted on experimental design, patient recruitment and selection, and running of the study. PHR consulted on experimental design and pathological interpretation. MWB consulted on experimental design and cognitive psychological interpretation.

## Conflict of Interest Statement

The authors declare that the research was conducted in the absence of any commercial or financial relationships that could be construed as a potential conflict of interest. The reviewer VP and handling Editor declared their shared affiliation, and the handling Editor states that the process nevertheless met the standards of a fair and objective review.

## Footnotes

^1^www.fmrib.ox.ac.uk/fsl
